# Synthesis and Preliminary Evaluation of a 2-Oxoquinoline Carboxylic Acid Derivative for PET Imaging the Cannabinoid Type 2 Receptor

**DOI:** 10.3390/ph7030339

**Published:** 2014-03-06

**Authors:** Linjing Mu, Roger Slavik, Adrienne Müller, Kasim Popaj, Stjepko Čermak, Markus Weber, Roger Schibli, Stefanie D. Krämer, Simon M. Ametamey

**Affiliations:** 1 Center for Radiopharmaceutical Sciences of ETH-PSI-USZ, Department of Nuclear Medicine, University Hospital Zürich, CH-8091 Zürich, Switzerland; 2 Center for Radiopharmaceutical Sciences of ETH-PSI-USZ, Institute of Pharmaceutical Sciences, Department of Chemistry and Applied Biosciences, ETH Zurich, CH-8093 Zürich, Switzerland; 3 Neuromuscular Diseases Unit/ALS Clinic, Kantonsspital St. Gallen, CH-9007 St. Gallen, Switzerland

**Keywords:** cannabinoid receptor type 2 ligand, CB2 receptor, neurodegeneration, radiolabeling, autoradiography, small-animal PET

## Abstract

Cannabinoid receptor subtype 2 (CB2) has been shown to be up-regulated in activated microglia and therefore plays an important role in neuroinflammatory and neurodegenerative diseases such as multiple sclerosis, amyotrophic lateral sclerosis and Alzheimer’s disease. The CB2 receptor is therefore considered as a very promising target for therapeutic approaches as well as for imaging. A promising 2-oxoquinoline derivative designated KP23 was synthesized and radiolabeled and its potential as a ligand for PET imaging the CB2 receptor was evaluated. [^11^C]KP23 was obtained in 10%–25% radiochemical yield (decay corrected) and 99% radiochemical purity. It showed high stability in phosphate buffer, rat and mouse plasma. In vitro autoradiography of rat and mouse spleen slices, as spleen expresses a high physiological expression of CB2 receptors, demonstrated that [^11^C]KP23 exhibits specific binding towards CB2. High spleen uptake of [^11^C]KP23 was observed in dynamic *in vivo* PET studies with Wistar rats. In conclusion, [^11^C]KP23 showed promising *in vitro* and *in vivo* characteristics. Further evaluation with diseased animal model which has higher CB2 expression levels in the brain is warranted.

## 1. Introduction

The use of cannabis as a therapeutic agent dates back about 5,000 years with descriptions of its numerous effects including alterations in mood, cognitive functions, memory and perception of the user [1]. The plant cannabis sativa, commonly known as marijuana contains over 60 compounds. The major psychoactive constituent is delta‐9‐tetrahydrocannabinol (Δ^9^‐THC). Cannabinoids exert their effects through an endogenous cannabinoid system in the central and peripheral nervous system. Currently two subtypes of cannabinoid receptors have been isolated and cloned: CB1 and CB2. There is also some evidence that other cannabinoid receptors GPR18 and GPR55 may exist [2,3]. Not much is known about GPR18 and GPR55 subtypes and current research efforts in the field of cannabinoid receptors are directed towards exploring their pharmacology and physiological roles. The CB1 receptor is the most studied receptor of the endocannabinoid system and is densely expressed in the CNS by many classes of neurons [4,5]. The CB2 receptor on the other hand is found predominantly in cells of the immune system, spleen, lymph nodes but has very low or undetectable expression levels in the CNS under basal conditions [6,7]. Under pathological conditions, however, the CB2 receptor can be up-regulated on activated microglia (macrophages of the brain). It is well known that cannabinoid receptors are involved in a broad range of processes including appetite, anxiety, memory, cognition, immune regulation and inflammation [8], however, the function of CB2 in neuroinflammation, microglia activation and intrusion of immune cells is not yet fully understood. Visualization and quantification of CB2 receptor expression by non-invasive PET imaging provides a high potential for understanding the role of CB2 in the development and progression of neuroinflammatory and neurodegenerative diseases.

In the past decade, substantial progress has been made in the synthesis and evaluation of radiotracers for PET imaging of the CB1 receptor [9,10,11]. [^18^F]MK-9470 has been used in the clinical setting to support CB1 receptor drug development for measuring receptor occupancy, to investigate CB1 receptor variability with gender and normal ageing and in studying pathophysiological conditions [12,13,14]. Although the pharmacological and therapeutic potential of selective CB2 ligands has been studied and reviewed extensively in the literature [15,16], the field of non-invasive CB2 imaging remains largely unexplored. Only a limited number of CB2 radioligands have been synthesized and tested as PET tracers [17,18,19,20,21], among them the 2-oxoquinoline-3-carboxamide derivative, [^11^C]NE40, which has been evaluated in healthy volunteers. [^11^C]NE40 exhibited the expected uptake in lymphoid tissue and appropriate brain kinetics [22]. Herein, we report the radiolabeling, *in vitro* and *in vivo* evaluation of a 2-oxoquinoline containing structure, [^11^C]KP23, as a potential PET radiotracer for imaging cannabinoid type 2 receptors.

## 2. Experimental Section

### 2.1. Materials and Methods

Animal experiments were in accordance with the Swiss Animal Welfare legislation and were approved by the Veterinary Office of the Canton Zurich. Six week old female NMRI mice and male Wistar rats were purchased from Charles River (Sulzfeld, Germany) and kept under standard conditions.

All chemicals, unless otherwise stated, were purchased from Sigma-Aldrich (Zug, Switzerland) or Merck (Buchs, Switzerland) and used without further purification. Solvents for extractions, column chromatography and thin layer chromatography (TLC) were purchased as commercial grade. Organic reactions were monitored by TLC analysis using Sigma-Aldrich silica gel 60 plates (2−25 μm). Mobile phase for TLC was a mixture of pentane and ethyl acetate at suitable ratios. Developed TLCs were visualized under UV light at 254 nm. Nuclear magnetic resonance (NMR) spectra (^1^H and ^13^C-NMR) were recorded in Fourier transform mode at the field strength specified on Bruker Avance FT-NMR spectrometers. The measured chemical shifts are reported in δ (ppm) and the residual signal of the solvent was used as the internal standard. Multiplicities in the ^1^H-NMR spectra are described as: s = singlet, d = doublet, t = triplet, m = multiplet, b = broad; coupling constants are reported in Hz. High resolution mass spectrometry (HRMS) was performed with a Bruker FTMS 4.7 T BioAPEXII spectrometer.

High-performance liquid chromatography (HPLC) analyses were performed using a reversed phase column (ACE column, C18, 3 µm). The mobile phase consisted of 0.1% TFA in water and acetonitrile. The acetonitrile gradient from 30% to 95% over 10 min at 1 mL/min flow rate was applied. Analytical radio-HPLC was performed on an Agilent 1100 series system equipped with a Raytest Gabi Star radiodetector (Agilent Technologies, Morges, Switzerland). Semi-preparative HPLC purifications were carried out using a reversed phase column (ACE column, Symmetry C8 5µm; 7.8 × 50 mm) under the following conditions: 0.1% H_3_PO_4_ in H_2_O (solvent A), MeCN (solvent B); 0.0–1.0 min, 30% B; 1.1–12.0 min, 30%–90% B; 12.1–20 min, 90% B; 20.1–40 min, 30% B; flow rate: 4 mL/min. A Merck-Hitachi L2130 system equipped with a radiation detector VRM 202 (Veenstra Instrument, Joure, the Netherlands) was used for semi-preparative HPLC. Specific activity was calculated by comparing ultraviolet peak intensity of the final formulated products with calibration curves of corresponding non-radioactive standards of known concentrations. For the *in vitro* and *ex vivo* stability studies, an Ultra-performance liquid chromatography (UPLC™) system from Waters with a Waters Acquity UPLC BEH C18 column (2.1 × 50 mm, 1.7 µm) and an attached Berthold co-incidence detector (FlowStar LB513, Berthold Technologies, Bad Wildbad,) was used. The mobile phase consisted of a gradient of acetonitrile in water with 0.1% TFA from 10% to 95% over 2 min, flow rate 0.6 mL/min.

### 2.2. Chemistry

#### 2.2.1. Synthesis of Compound 7 as Precursor for ^18^F-Radiolabeling

To a solution of 8-butoxy-*N*-(2-hydroxy-2-phenylethyl)-7-methoxy-2-oxo-1,2-dihydroquinoline-3-carboxamide (**6**, 615 mg, 1.5 mmol) in dichloromethane (DCM, 5 mL) was added triethylamine (300 mg, 3.0 mmol). The mixture was cooled to 0 °C in an ice-bath and a solution of methanesulfonyl chloride (205 mg, 1.8 mmol) in DCM (3 mL) was added dropwise. The mixture was stirred over night at RT. The reaction mixture was diluted with EtOAc (200 mL) and washed with 0.2 M aq. HCl (3 × 20 mL) and brine (2 × 10 mL). The organic layer was dried over Na_2_SO_4_ and solvents were removed under reduced pressure. The crude was purified over 100 g silica gel using EtOAc/Hexan 1:1 afforded 8-butoxy-*N*-(2-chloro-2-phenylethyl)-7-methoxy-2-oxo-1,2-dihydroquinoline-3-carboxamide (**7**, 263 mg, 0.614 mmol, 41% yield), but not the expected mesylated product. ^1^H-NMR (400 MHz, CDCl_3_): δ 10.00 (t, *J* = 5.7 Hz, 1H), 9.30 (s, 1H), 8.85 (s, 1H), 7.48–7.44 (m, 3H), 7.40–7.31 (m, 3H), 6.94 (d, *J* = 4.5 Hz, 1H), 5.15 (dd, *J_1_* = 5.4 Hz, *J_2_* = 8.5 Hz, 1H), 4.18–4.07 (m, 3H), 3.97 (s, 3H), 3.94–3.87 (m, 1H), 1.83–1.76 (m, 2H), 1.55–1.46 (m, 2H), 0.99 (t, *J* = 7.4 Hz, 3H). HRMS: calculated for [M+H]^+^ C_23_H_26_ClN_2_O_4_ is 429.1581; found 429.1576; Purity by HPLC: 98%.

#### 2.2.2. Synthesis of Compound KP26 as Precursor for ^11^C-Radiolabeling

To a solution of 8-butoxy-*N*-(2-fluoro-2-phenylethyl)-7-methoxy-2-oxo-1,2-dihydroquinoline-3-carboxamide (KP23, 660 mg, 1.6 mmol) in DMF (15 mL) was added lithium chloride (1.36 g, 32 mmol). The mixture was heated to reflux for 4 hours. After cooling to RT, the mixture was diluted with EtOAc (400 mL) and washed with aq. HCl (0.2 M, 3 × 30 mL) and brine (2 × 30 mL) and dried over Na_2_SO_4_. Solvents were removed under reduced pressure and column chromatography of the crude over 200 g silica gel using EtOAc/hexane/EtOH 1:1:0.1 gave pure 8-butoxy-*N*-(2-fluoro-2-phenylethyl)-7-hydroxy-2-oxo-1,2-dihydroquinoline-3-carboxamide (KP26, 458 mg, 1.150 mmol, 71.8% yield). ^1^H-NMR (400 MHz, CDCl_3_): δ 9.91 (s, 1H), 9.23 (s, 1H), 8.84 (s, 1H), 8.50 (br. s, 1H), 7.34 (d, *J* = 8.7 Hz, 1H), 7.28–7.22 (m, 1H), 7.05–7.00 (m, 2H), 6.98–6.95 (m, 1H), 6.90 (dt, *J_1_* = 2.1 Hz, *J_2_* = 8.4 Hz, 1H), 4.13 (t, *J* = 6.9 Hz, 2H), 3.77–3.71 (m, 2H), 2.95 (t, *J* = 7.2 Hz, 2H), 1.83–1.76 (m, 2H), 1.52–1.43 (m, 2H), 0.96 (t, *J* = 7.4 Hz, 3H). ^13^C-NMR (100 MHz, CDCl_3_): δ 164.3, 162.1, 145.4, 133.9, 131.0, 130.0, 129.9, 125.9, 124.5, 124.4, 115.8, 115.5, 114.3, 113.58, 113.5, 113.3, 73.8, 41.0, 35.5, 32.2, 19.1, 13.8. HRMS: calculated for [M + H]^+^ C_22_H_24_N_2_O_4_ is 399.1715; found 399.1711; Purity by HPLC: 98%.

### 2.3. Radiochemistry

#### 2.3.1. Radiosynthesis of [^18^F]KP23

No-carrier-added (n.c.a) [^18^F]fluoride was produced via the ^18^O(p,n)^18^F nuclear reaction using an IBA Cyclone 18/9 cyclotron (IBA, Ottignies-Louvain-la-Neuve, Belgium). For this, >98% isotopically enriched ^18^O-water (Nukem GmbH, city, Germany) was irradiated by 18 MeV proton beams. Produced [^18^F]fluoride/[^18^O]water solution was transferred using a helium stream from the target to a shielded hot cell equipped with a manipulator where radiosynthesis was performed. The activity was trapped on a QMA cartridge (preconditioned with 0.5 M aq. K_2_CO_3_ (1 × 5 mL) and then H_2_O (1 × 5 mL) and dried in air. The trapped [^18^F]fluoride was eluted from the cartridge and eluted with a solution of Kryptofix (K_2.2.2_, 5 mg) and K_2_CO_3_ (1 mg) in acetonitrile (1.4 mL) and water (0.6 mL) or tetrabutylammonium hydroxide (0.6 mL, 0.18 mM) into a 10 mL sealed reaction vessel. The [^18^F]fluoride (*ca.* 10−15 GBq) was dried by azeotropic distillation of acetonitrile at 110 °C under vacuum with a stream of nitrogen. The azeotropic drying process was repeated 3 times with 1 mL of acetonitrile. To the dried K_2.2.2_/K[^18^F]F complex was added the chlorinated compound **7** (*ca.* 2 mg) in different anhydrous solvents such as DMF, DMSO or acetonotrile (0.3 mL), and the reaction mixture was heated and analyzed by HPLC.

#### 2.3.2. Radiosynthesis of [^11^C]KP23

Carbon-11 was produced via the ^14^N(p,α)^11^C nuclear reaction at a Cyclone 18/9 cyclotron (18 MeV, IBA) in the form of [^11^C]CO_2_. [^11^C]Methyl iodide ([^11^C]MeI) was generated in a 2-step reaction sequence involving the catalytic reduction of [^11^C]CO_2_ to [^11^C]methane and subsequent gas phase iodination. [^11^C]KP23 was prepared by reaction of the desmethyl precursor compound (0.5–1 mg) with [^11^C]MeI (*ca.* 40–50 GBq) in DMF solution in the presence of Cs_2_CO_3_ (5 mg) at 120 °C for 3 min. The crude product was diluted with water (1.3 mL) and injected onto a semi-preparative HPLC. The radiolabeled product was collected, diluted with water (10 mL), passed through a preconditioned C18 cartridge (Waters, Boston, MA, USA, preconditioned with 5 mL EtOH and 10 mL water), washed with water (5 mL), and eluted with EtOH (0.5 mL). After adding 9.5 mL of water, the 5% ethanol solution containing [^11^C]KP23 was then passed through a sterile filter (0.2 µm) and used for all *in vitro*/*in vivo* studies.

### 2.4. *In Vitro* Characterization

#### 2.4.1. Competition Binding Assay

Frozen membrane preparations from CHO-K1 cells transfected with human CB1 (hCB1) and CB2 (hCB2), respectively (PerkinElmer, Waltham, MA, USA), were thawed on ice and diluted to a final protein concentration of 1 µg/mL in assay buffer (50 mM TRIS/HCl, 1 mM EDTA (Applichem, Darmstadt, Germany), 3 mM MgCl_2_, pH 7.4, containing 0.05% bovine serum albumin, BSA). Membrane dilutions (0.5 µg protein in 550 µL final volume) were incubated at 30 °C with KP23 at concentrations between 10^−5^ and 10^−11^ M and 1.4 nM [^3^H]CP-55,940 (PerkinElmer, Waltham, MA, USA), a hCB1 and hCB2 agonist with K_i_ values of 0.58 and 0.68 nM, respectively. Nonspecific binding of [^3^H]CP-55,940 was determined after addition of 5 μM hCB1/hCB2 agonist WIN-55212-2 (K_i_ 1.89 and 0.28 nM, respectively) [23]. All samples were prepared in triplicates. After 90 min, 3 mL ice cold assay buffer was added and samples were immediately filtered through Whatman GF/C filters (pre-soaked in 0.05% polyethylenimine) and washed twice with 3 mL ice cold assay buffer. Bound [^3^H]CP-55,940 was quantified in a Beckman LS 6500 Liquid Scintillation Counter (Beckman^TM^, Brea, CA, USA) and IC_50_ values were determined by non-linear regression analysis [24]. K_i_ values were determined with the equation from Cheng-Prusoff and are means from three independent experiments (K_D_ values of 0.14 and 0.11 nM from PerkinElmer were used for [^3^H]CP-55,940 binding to hCB1 and hCB2 receptors, respectively).

#### 2.4.2. *In Vitro* Stability Studies

To test the plasma stability of the radioligand, 10 µL (4 MBq) of [^11^C]KP23 solution were added to phosphate buffer (300 µL, 4 mM NaH_2_PO_4_/Na_2_HPO_4_, 155 mM NaCl, pH 7.4), mouse and rat plasma (300 µL), respectively. The mixture was vortexed and four aliquots of 70 µL were incubated at 37 °C. Ice cold acetonitrile (100 µL) was added to stop the enzymatic reactions at different time points (0, 10, 20 and 40 min). After centrifugation (10,000 *g*, 3 min), the supernatant was passed through a filter (0.45 µm, Minisart SRP 4, Sartorius Stedim Biotech, Goettingen, Germany) and analyzed by UPLC.

#### 2.4.3. *In Vitro* Autoradiography

*In vitro* autoradiography with [^11^C]KP23 was performed on 20 µm tissue slices (rat/mouse spleen) adsorbed to SuperFrost Plus slides. Slices were thawed (10 min) on ice before pre-incubation with incubation buffer (50 mM TRIS/HCl, pH 7.4, containing 5% BSA) at 4 °C for 10 min. Excess solution was carefully removed and the tissue slices were dried in a ventilated hood for 10 min. Then slides were incubated with 0.6 or 0.2 nM [^11^C]KP23 alone or together with specific CB2 agonist (GW405833, 1 µM) in incubation buffer. After incubation for 15 min at RT in a humid chamber, slides were washed twice with ice cold washing buffer (50 mM TRIS/HCl, pH 7.4, 1% BSA, 5% EtOH) for 2 min each and dipped twice in water. Dried slides were exposed to a phosphor imager plate for 30 min and the plate was scanned in a BAS5000 reader (Fujifilm, Dielsdorf, Switzerland).

### 2.5. *In Vivo* Characterization

#### 2.5.1. *In Vivo* PET Imaging

PET scanning was performed with the GE VISTA eXplore PET/CT tomograph (Sedecal, Madrid, Spain). The scanner is characterized by high sensitivity (absolute central point source sensitivity of 4% for the 250–700 eV energy windows) and an axial field of view of 4.8 cm [25]. Male Wistar rats (278 ± 2 g, n = 2) were immobilized by isoflurane inhalation and the tracer was injected into a tail vein on the tomographic bed [26]. Tracer accumulation in the spleen or brain were recorded by dynamic one-bed position PET scans over 60 min starting with the injection of 13 respectively 40 MBq (0.05 to 0.15 nmol) of [^11^C]KP23. PET data was reconstructed by 3-dimensional FORE/2-dimensional OSEM in user-defined time frames with a voxel size of 0.3875 × 0.3875 × 0.775 mm. Singles and random corrections but no attenuation correction were applied. Image files were evaluated with the software PMOD v3.4 (PMOD Technologies Inc., Zurich, Switzerland). Time activity curves (TACs) of brain regions were generated with the implemented rat brain region of interest (ROI) template and TACs of the abdominal region with the respective ROIs generated by the PMOD segmentation tool. Spleen, liver and background TACs were confirmed by analysis of manually drawn regions of interest. Standardized uptake values (SUV) were calculated as tissue activities (Bq/cm^3^), normalized to the injected dose per body weight (Bq/g).

#### 2.5.2. Metabolite Studies

[^11^C]KP23 was administered intravenously to a restrained Wistar rat (413 g) by tail vein injection (*ca.* 160 MBq). The animal was sacrificed by decapitation 30 min p.i. and blood and urine were collected. Whole blood was collected in heparin-coated tubes (BD Vacutainers, Plymouth, UK) and centrifuged at 5,000 *g* for 5 min, 4 °C. The proteins of the supernatant plasma and urine were precipitated by addition of an equal volume of acetonitrile and centrifuged at 5,000 *g* for another 5 min. The supernatants were filtered and analyzed by UPLC. For the determination of *in vivo* radiometabolites at 15 min p.i. blood samples were withdrawn from the tail vein opposite to the injection site.

## 3. Results and Discussion

### 3.1. Chemistry and Radiochemistry

The non-radioactive standard reference compound KP23 was synthesized based on the modified procedure described by Turkman [27]. Compound **2** was obtained in 30% yield by the aromatic nitration of 3-hydroxy-4-methoxybenzaldehyde (**1**) with nitric acid. Then *O*-alkylation with 1-bromobutane afforded compound **3** in 89% yield. Reduction of compound **3** using iron powder followed by reaction *in situ* with dimethyl malonate yielded compound **4**. The acid compound **5** was produced quantitatively by saponification reaction of the methyl ester compound **4** under basic conditions. Amide bond formation using the acid compound **5** with either 2-amino-1-phenylethanol or 2-fluoro-2-phenylethanamine free base led to compound **6** and KP23, respectively. Compound **6** was reacted with mesylchloride to afford chloro derivative **7**, which was used as supposed to be a precursor for fluorine-18 labeling. Demethylation of compound KP23 was performed with LiCl to afford the precursor compound KP26 for carbon-11 labeling. The newly synthesized compounds were characterized by mass spectrometry and NMR and their chemical purities were assessed by HPLC.

**Scheme 1:**
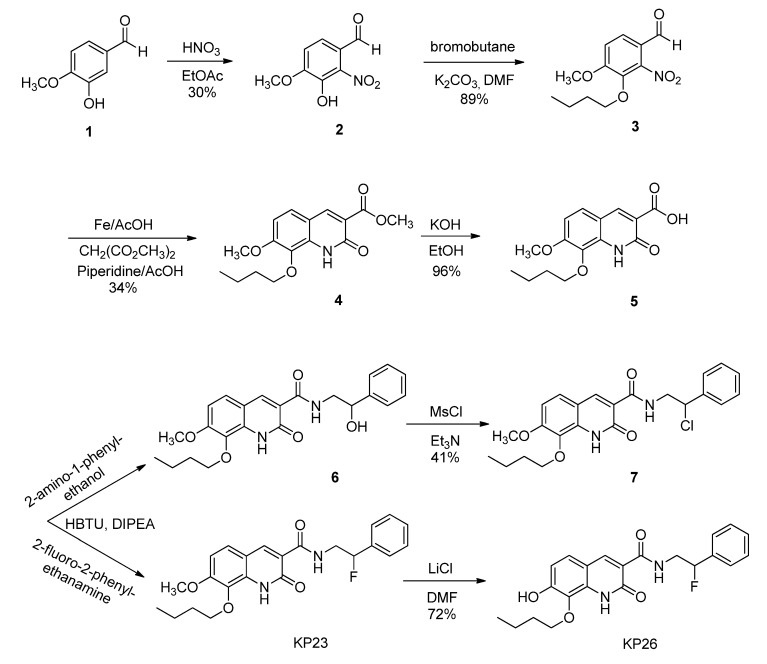
Synthesis of the standard reference KP23 and precursors **7** and KP26 for radiolabeling.

**Scheme 2:**
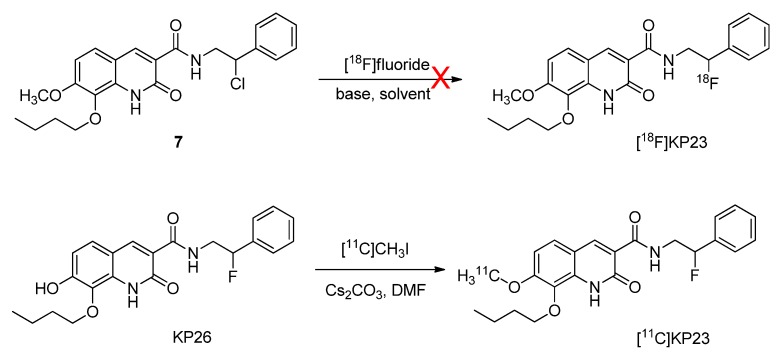
Radiosyntheses of compounds [^18^F]KP23 and [^11^C]KP23.

The structure of compound KP23 (Scheme 1) is amenable to radiolabeling with either fluorine-18 or carbon-11 (Scheme 2), therefore, the corresponding chloro and phenolic precursor compounds **7** and KP26 were prepared and tested for radiolabeling. For the fluorine-18 radiolabeling of KP23, it was originally planned to synthesize a precursor with a sulfonate leaving group. Initial attempts to tosylate compound **6** to afford a precursor with a tosyl leaving group were unsuccessful. Mesylation, however, exclusively afforded chloro analog **7** in 41% yields. This result has also been observed for other similar compounds in the literature [28,29]. It is suggested that this conversion occurs via spontaneous displacement of the sulfonate group by nitrogen (anchimeric assistance) with formation of reactive aziridinium ion intermediate. Consequent collapse of the aziridinium ion pair to the more stable chloride then occurs, upon exchange of the mesylate counterion with chloride [30]. ^18^F-radiolabeling was unsuccessful even using different solvents e.g., MeCN, DMF or DMSO and combined with different [^18^F]-reagents such as K_2.2.2_/K[^18^F]F or [^18^F]TBAF. Neither increasing the reaction temperature, duration of the radiolabeling reaction nor precursor concentrations had any positive influence on the outcome of radiolabeling yield. This can be explained by the fact that chloride is a poor leaving group.

KP23 was successfully labeled with carbon-11 in a one-step reaction by reacting the phenolic precursor with [^11^C]methyl iodide (Scheme 2). [^11^C]KP23 (*ca.* 1–3 GBq) was obtained in 99% radiochemical purity after semi-preparative HPLC purification. The total radiolabeling time was around 40 min after delivery of [^11^C]CO_2_ from the cyclotron to the hot-cell. Specific activity was high and ranged from 80 to 240 GBq/µmol at the end of synthesis. The radiochemical yield was 10%–25% (decay corrected).

### 3.2. *In Vitro* Characterization

**Figure 1:**
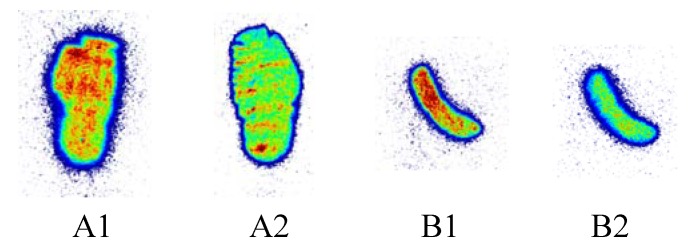
Autoradiography of rat (**A**) and mouse (**B**) spleen sections incubated with 0.6 nM [^11^C]KP23 in the absence of blocking agent (A1 and B1) or in the presence of excess blocking agent 1 μM GW4058233 (A2 and B2).

In vitro competitive binding assays were performed with membranes obtained from CHO-K1 cells stably transfected with human CB1 and CB2, respectively, using [^3^H]-CP-55940. The binding affinity of the nonradioactive KP23 obtained from three independent experiments was 6.8 ± 5.8 nM towards human CB2 and > 10,000 nM towards CB1, slightly lower affinity than the reported data of 0.8 ± 0.3 nM towards human CB2 and >10,000 nM towards CB1 [27]. The affinity and selectivity of KP23 towards CB2 are the highest among the known 2-oxoquinoline carboxylic acid derivatives including [^11^C]NE40, the first CB2 PET tracer evaluated in healthy human subjects which exhibits a K_i_ value of 9.6 nM. Autoradiography with slices from rat and mouse spleen, an organ with high CB2 levels [31], demonstrated high binding which was blocked by excess GW4058233 (CB2 specific agonist), indicating specific binding of [^11^C]KP23 to CB2 (Figure 1) in both cases. However, relatively high non-specific binding was observed in the spleen tissue (Figure 1 A2 and B2) which is not surprising considering the calculated LogP value of 3.81.

**Figure 2:**
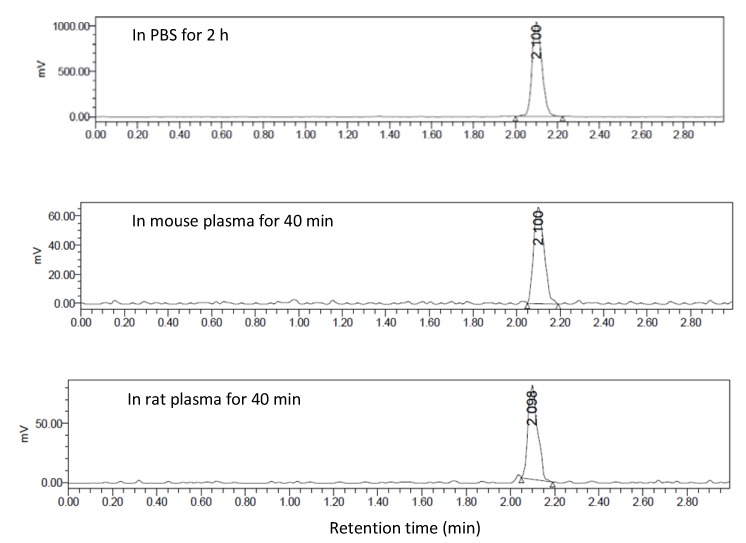
Radio-UPLC profiles of [^11^C]KP23 (t_R_ = 2.1 min) after 2 h in PBS and after 40 min in mouse and rat plasma.

*In vitro* plasma stability tests were carried out in PBS, mouse and rat plasma over a period of 40 min at 37 °C. No radioactive degradation products of [^11^C]KP23 were detected (Figure 2).

### 3.3. *In Vivo* PET Imaging of [^11^C]KP23

**Figure 3:**
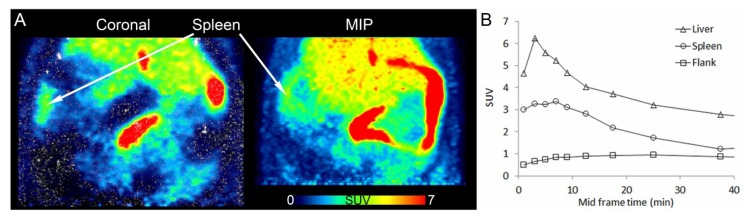
PET/CT images of [^11^C]KP23 in the spleen-liver region in rat. (**A**) Coronal section and maximal intensity projection (MIP) averaged from 6 to 15 min p.i. (**B**) TACs of [^11^C]KP23 in spleen, liver and flank.

Figure 3A shows PET images of a coronal section and maximal intensity projection (MIP) of the abdominal region of a rat fused to the respective CT, and averaged from 6 to 15 min p.i. [^11^C]KP23 accumulated in spleen, liver and intestines. The high spleen uptake is in accordance with the expression pattern of CB2 while the high accumulation in liver suggests a hepatobiliary elimination pathway. Background radioactivity in muscle tissue was relatively low. The TACs of liver, spleen and peripheral tissue outside the rips are shown in Figure 3B. Radioactivity peaked around 4 min p.i. for liver; the level of activity concentration steadily increased during the initial 8 min p.i. in spleen and then slowly washed out. During the whole data acquisition, the level of activity concentration was higher in the spleen region than in the background (flank).

**Figure 4:**
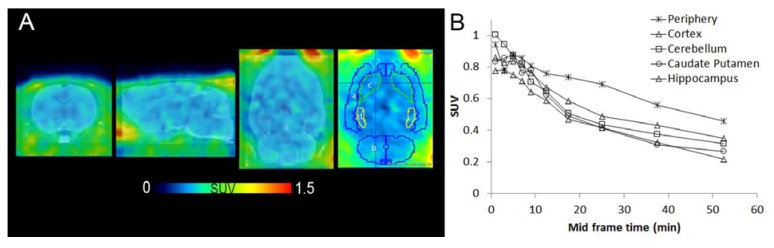
[^11^C]KP23 PET images of a rat brain superimposed on an MRI template . A: From left, axial, sagittal and coronal sections. In the last coronal image, regions of interest are encircled and used for TAC analysis shown in B. a, cortex; b, cerebellum; c, caudate putamen; d, hippocampus. Images are averaged from 2–60 min p.i. B TACs of rat brain regions of [^11^C]KP23.

Distribution of the tracer in rat brain was evaluated by PET (Figure 4A). Region of interest TAC analysis showed low uptake in healthy rat brain which is in line with the low expression level of CB2 receptor (Figure 4B). Radioactivity levels in brain were lower than in peripheral tissues in general. The expression profile of CB2 provides great opportunities for PET imaging with low background in the brain region, whereas under inflammation conditions a 10-100-fold higher CB2 receptor density is expected in activated microglia [32].

### 3.4. *In Vivo* Metabolic Studies

**Figure 5:**
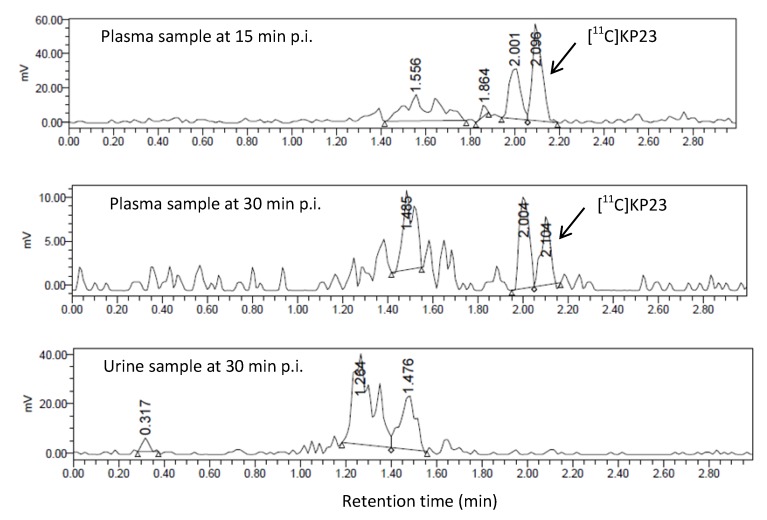
Radio-UPLC profiles of [^11^C]KP23 metabolites in blood plasma samples obtained at 15 min and 30 min p.i. and in the urine sample obtained at 30 min p.i.

The metabolic fate of [^11^C]KP23 was studied using blood and urine samples of a Wistar rat. In blood plasma samples, two to three radiometabolites which were more hydrophilic than the parent tracer, [^11^C]KP23, were detected. As illustrated in Figure 5, one major radiometabolite slightly more polar than the parent compound was generated in blood plasma samples. The percentage of parent tracer radioactivity decreased to 40% at 15 min p.i., 25% at 30 min p.i. No intact compound could be found in the urine sample after 30 min p.i.

## 4. Conclusions

[^11^C]KP23 showed promising *in vitro* characteristics. Its affinity and selectivity for CB2 is the highest among the known 2-oxoquinoline carboxylic acid derivatives. In vitro autoradiography with slices from rat and mouse spleen demonstrated specific binding. High spleen uptake of [^11^C]KP23 was also observed in dynamic PET studies with Wistar rats. As expected, distribution to healthy rat brain was relatively low in the *in vivo* PET experiments. Further evaluation of [^11^C]KP23 using neuroinflammation animal models which has higher CB2 expression levels in the brain is warranted.
